# Status of malaria in pregnancy services in Madagascar 2010–2021: a scoping review

**DOI:** 10.1186/s12936-023-04497-3

**Published:** 2023-02-20

**Authors:** Ashley Malpass, Natasha Hansen, Catherine M. Dentinger, Susan Youll, Annett Cotte, Chiarella Mattern, Aimée Ravaoarinosy

**Affiliations:** 1 US President’s Malaria Initiative, USAID, Washington, DC USA; 2 Malaria Branch, US President’s Malaria Initiative, Centers for Disease Control and Prevention, Atlanta, GA USA; 3 Institut Pasteur de Madagascar, Antananarivo, Madagascar; 4 National Malaria Control Programme, Ministry of Health, Antananarivo, Madagascar

**Keywords:** Malaria, Madagascar, IPTp, Pregnancy

## Abstract

**Background:**

Malaria in pregnancy (MIP) increases the risk of poor maternal and infant outcomes. To reduce these risks, WHO recommends insecticide-treated net (ITN) use, intermittent preventive treatment during pregnancy (IPTp) with sulfadoxine-pyrimethamine (SP), and prompt case management. However, uptake of these interventions remains sub-optimal in Madagascar. A scoping review was conducted to determine the breadth and depth of information available during 2010–2021 about Madagascar’s MIP activities and to identify barriers and facilitators to MIP interventions uptake.

**Methods:**

PubMed, Google Scholar, and USAID’s files (Development Experience Catalog) were searched using the terms “Madagascar AND pregnancy AND malaria,” and reports and materials from stakeholders were collected. Documents in English and French from 2010 to 2021 with data regarding MIP were included. Documents were systematically reviewed and summarized; results were captured in an Excel database.

**Results:**

Of 91 project reports, surveys and published articles, 23 (25%) fell within the stated time period and contained relevant data on MIP activities in Madagascar and were categorized accordingly: eight (35%) quality of care, including health facility readiness, provider knowledge and commodity availability; nine (39%) care-seeking behaviour; and, six (26%) prevention of MIP. Key barriers were identified: nine articles mentioned SP stockouts; seven found limitations of provider knowledge, attitudes, and behaviours (KAB) regarding MIP treatment and prevention; and, one reported limited supervision. MIP care seeking and prevention barriers and facilitators included women’s KAB regarding MIP treatment and prevention, distance, wait times, poor service quality, cost, and/or unwelcoming providers. A 2015 survey of 52 health facilities revealed limited client access to antenatal care due to financial and geographic barriers; two 2018 surveys revealed similar findings. Self-treatment and care-seeking delays were reported even when distance was not a barrier.

**Conclusion:**

Among the studies and reports on MIP in Madagascar, the scoping review frequently noted barriers that could be mitigated by reducing stockouts, improving provider knowledge and attitudes, refining MIP communication, and improving service access. There is a need for coordinated efforts to address the identified barriers is the key implication of the findings.

## Background

Malaria in pregnancy (MIP) is a serious condition for both mother and fetus that can lead to anaemia, low birthweight, stillbirth, and maternal death [1]. To reduce maternal and fetal morbidity and mortality from malaria, the World Health Organization (WHO) recommends pregnant women living in malaria-endemic areas sleep under an insecticide-treated net (ITN), take intermittent preventive treatment (IPTp) with sulfadoxine-pyrimethamine (SP) monthly beginning in the second trimester of pregnancy, and receive prompt diagnosis and effective treatment for malaria [2]. However, uptake of these measures across much of sub-Saharan Africa remains below national targets in part due to limited coordination between malaria control and mother and child health programmes, out-of-date policies, and commodity supply shortages [3, 4].

The entire population of Madagascar is at risk of malaria, and the reported incidence ranged from < 1 to 100 cases per 1,000 population across the island nation in 2021 [5]. Madagascar recommends antenatal care (ANC) for all pregnant women in the country beginning in the first trimester of pregnancy, and prompt care seeking for febrile illness. Additionally, in 106 of 114 health districts of the country where the annual malaria incidence is > 1/1000 population, it is recommended that pregnant women consistently sleep under an ITN and receive IPTp. Current MIP prevention policies include providing an ITN at the first ANC visit with instruction on proper use; initiating IPTp as early in the second trimester as possible and continuing through delivery; prompt diagnosis and effective treatment of malaria during pregnancy; and behaviour change communication strategies to reinforce these interventions [6].

For IPTp, SP is administered as directly observed therapy (DOT) every four weeks starting at 13 weeks of gestational age. IPTp is delivered during ANC visits in health facilities by a health care worker ((HCW), nurse, midwife, or physician). In three pilot districts, Madagascar is testing IPTp delivered at the community level by trained community health volunteers (CHVs). This pilot, Transforming Intermittent Preventive Treatment for Optimal Pregnancy (TIPTOP), is designed to investigate the impact of community-IPTp on improving access and uptake [7]. In addition, HCWs and CHVs promote ANC attendance and encourage prompt care seeking for febrile illness among all pregnant women [6].

Data to determine MIP burden is limited in Madagascar. The Madagascar National Malaria Control Programme (NMCP) in collaboration with other Ministry of Health departments, sets targets for ANC attendance, IPTp uptake, and ITN coverage and use, and monitors progress toward achieving these targets. IPTp indicators include the proportion of recently pregnant women who initiated IPTp (IPTp1) and the number who completed three courses (IPTp3) used to estimate IPTp coverage. Population-based household surveys, such as the Malaria Indicator Survey (MIS), include self-reports of ITN and IPTp use, and indicate low uptake of both interventions. The most recent Madagascar MIS, conducted in 2016, revealed that 69% of pregnant women reported sleeping under an ITN the previous night, and 10% of women who had given birth in the previous two years reported taking a minimum of three doses of IPTp [8]. More recent estimates of IPTp3 uptake from project data range from 33% in 2017 [6] to 57% in select regions in 2020 [7], well below the country’s target of 80% [6].

As a first step toward improving MIP services in Madagascar, the NMCP, US President’s Malaria Initiative (PMI), and the Institut Pasteur de Madagascar (IPM) conducted a scoping review to determine what available information existed regarding MIP activities in Madagascar and to identify barriers and facilitators of ANC attendance and IPTp uptake. A scoping review was selected as the most appropriate methodology due to the limited number of articles in the published literature, and the potentially substantial information in unpublished project reports and surveys.

## Methods

This work was guided by the methodological framework for scoping reviews developed by Arksey and O’Malley [9], and refined by others [10, 11].

The purpose of this review was to determine the breadth and depth of available information on MIP activities in Madagascar and to describe the status of MIP prevention and treatment activities, including ANC attendance, IPTp uptake, ITN use and prompt care seeking among pregnant women.

### Document identification

Studies and reports on maternal health care in Madagascar were collected by searching databases and inquiries regarding non-published articles to key informants, which included members of the NMCP and partner organizations. For published articles, PubMed and Google Scholar were searched using the terms “malaria AND pregnancy AND Madagascar” for the years 2010–2021. The same terms and time frame were used to search for reports in the US Agency for International Development (USAID)’s database: the Development Clearing House (DEC). References in these documents were reviewed for additional relevant materials. Key informants were contacted via email regarding relevant reports, surveys and studies that may not have been in searchable databases. Titles and abstracts of materials identified during the search were compiled in an Excel database and screened by two reviewers. Only titles and abstracts with information on MIP in Madagascar were included for further review. If publications or final reports were available, interim reports of the same project were excluded. Only materials available in English or French were reviewed; if both English and French versions of a document were available, only the English version was included.

### Document review procedures

Documents were reviewed for relevant content and grouped as: care seeking; MIP prevention; and, quality of care, including health facility/health care worker capacity for providing MIP services. Documents that did not include information on any components of MIP prevention or treatment specifically in Madagascar were excluded. A data extraction tool based on PRISMA (Preferred Reporting Items for Systematic reviews and Meta-Analyses) extension guidelines for scoping reviews was designed to capture and summarize relevant findings [11]. For each document, information including title, date of activity, timeframe, implementer or author, publication source, language, and type of study or activity were recorded. The MIP-specific data elements gathered and considered essential for a comprehensive MIP programme were number of ANC visits; doses of IPTp; knowledge, attitudes, and beliefs regarding MIP prevention and treatment among HCWs, CHVs and pregnant women; availability of SP; and coordination of MIP activities between NMCP and Maternal and Child Health (MCH) programmes, as MIP programmes are implemented through the ANC platform. Documents focusing primarily on IPTp coverage, ITN use, and factors associated with women receiving IPTp or using ITNs were classified as prevention of MIP. The quality of care grouping contained information on provider knowledge, provider behaviour, and facility readiness, including availability of MIP commodities. In some documents, only a per cent or a numerator value was mentioned; thus only these values are reported in the results. For qualitative works, key results were documented.

## Results

A total of 91 documents were found for the period 2010–2021; these included articles published in peer reviewed journals, PowerPoint presentations, infographics, and reports from USAID-funded projects. Dissertations from Madagascar universities were not searchable, and a review of article citations did not produce any additional materials. Public documents and poster presentations were shared through key informant consultations. Of the 91 documents, 23 (25%) met the inclusion criteria. Of the included documents, six (26%) were peer-reviewed publications, 16 (70%) were partner reports, and one was an infographic (4%) (Table 1). Included documents were categorized as focusing primarily on prevention of malaria during pregnancy (6 (26%)), quality of care including facility readiness (8 (35%)), and care seeking (9 (39%)). Information about case management of MIP as well as coordination between NMCP and MCH was not found in any of the reviewed documents.

**Table 1:** Included articles by year, author, document type, and category, Malaria in Pregnancy Scoping Review, Madagascar, 2010–2020

Document title	Author/implementer	Year	Type of document	Category
Attitudes, beliefs, and practices relevant to malaria prevention and treatment in Madagascar [34]	Johns Hopkins Center for Communication Programs	2014	Partner report	Prevention of MIP: ANC1, IPTp1-2, KABW
Coverage of intermittent preventive treatment of malaria in pregnancy in four sub-Saharan countries: findings from household surveys [15]	Pons-Duran et al	2021	Peer reviewed journal article	Care Seeking: ANC1, ANC1 + , ANC8, IPTp1-3 +
*Etude de la situation de référence du Programme de santé ACCESS* [22]	Management Sciences for Health	2019	Partner report	Care seeking: ANC, ANC4 + , ANC8, IPTp1-3 + , KABW
*Etude des determinants du Recours aux Soins des femmes enceintes et des enfants de moins de 5 ans* [25]	Institut Pasteur de Madagascar	2019	Partner report	Care seeking: ANC1, ANC4 + , KABW
Evaluation of community delivery of Malaria Intermittent Preventive Treatment in Pregnancy (C-IPTp) (TIPTOP project) MIDLINE CLUSTER HOUSEHOLD SURVEY REPORT 2019—2020 MADAGASCAR, R.D. CONGO, NIGERIA AND MOZAMBIQUE [13]	TIPTOP	2020	Partner report	Quality of Care: ANC4 + , ANC8, IPTp3 +
Evaluation of community delivery of Malaria Intermittent Preventive Treatment in Pregnancy (C IPTp): Results of the 3rd Phase Data Collection Qualitative Research [35]	TIPTOP	2021	Partner report	Care Seeking: ANC1, KABW, SO
*Experiences et perceptions sur la recherche de soins pour les maladies febriles chez les gardes d'enfants de moins de 15 ans incluant les femmes enceintes et les prestataires de soins de santé dans 8 districts de Madagascar* [19]	MSCP	2017	Partner report	Care seeking: KABW, KABP, SO
Factors influencing the uptake of intermittent preventive treatment among pregnant women in sub-Saharan Africa: a multilevel analysis [17]	Darteh et al	2021	Peer reviewed journal article	Care Seeking: ANC1 IPTp2, IPTp3 +
IMPACT quarterly report 2, FY2020, Q2 [36]	PATH	2020	Partner report	Care seeking: ANC1, IPTp3 + , KABW, SO
Improving malaria prevention, diagnosis and treatment by investing in supply chains: support under the USAID | DELIVER PROJECT [27]	John Snow, Inc	2017	Partner report	Quality of Care: PTp2, SO
Madagascar overview Mananjary district: 2017 rapid facility assessment [29]	TIPTOP	2017	Infographic	Quality of Care: KABP, SO
Malaria-related ideational factors and other correlates associated with intermittent preventive treatment among pregnant women in Madagascar [14]	Awantag et al	2018	Peer reviewed journal article	Prevention of MIP: ANC1, ANC4 + , IPTp2, KABW, SO
Maternal and child survival program in Madagascar baseline report [21]	MCSP	2015	Partner report	Quality of Care: SO
MCSP Madagascar technical brief improving the prevention and management of malaria during pregnancy [4]	MSCP	2019	Partner report	Quality of Care: ANC8, IPTp3 + , KABP, SO
Nationwide evaluation of malaria infections, morbidity, mortality, and coverage of malaria control interventions in Madagascar [12]	Kesteman et al	2014	Peer reviewed journal article	Prevention of MIP: IPTp2-3 +
Preventing malaria during pregnancy in SSA Determinants of Effective IPTp [16]	Florey et al	2013	Partner report	Quality of Care: ANC1, ANC4 + , IPTp1-2
Surveillance and data for management (SDM) project final report [37]	Institut Pasteur de Madagascar	2020	Partner report	Prevention of MIP: IPTp1
*Tazomoka* is not a problem [23]	Mattern et al	2016	Peer reviewed journal article	Care seeking: KABP
Toolkit to improve early and sustained intermittent preventive treatment in pregnancy uptake: results from a formative evaluation in Madagascar and Mozambique [28]	Jhpiego	2017	Partner report	Quality of Care: KABP
Trust, community health workers and delivery of intermittent preventive treatment of malaria in pregnancy: a comparative qualitative analysis of four sub-Saharan countries [30]	Enguita-Fernàndez et al	2020	Peer reviewed journal article	Care seeking: KABW
USAID Community Capacity for Health Program—Mahefa Miaraka, FY2017 annual report, october 1, 2016 to september 30, 2017 [18]	John Snow, Inc	2017	Partner report	Prevention of MIP: ANC1, ANC4 + , IPTp2 +
USAID/Madagascar Health Survey, in the North and West Regions of the Community Capacity for Health (CCH) intervention zones [38]	Meekers et al	2017	Partner report	Prevention of MIP: ANC4 + , IPT1-2, KABP
Use of malaria in pregnancy facility assessment tool to identify key challenges in implementing IPTP in Moramanga District Madagascar [26]	Henry et al	2018	Poster presentation	Quality of Care: KABP, SO

*ANC1* at least one antenatal care visit; *ANC4* + four or more antenatal visits; *ANC8* WHO recommendation for 8 antenatal contacts; *IPTp1-3* + intermittent preventive treatment of malaria in pregnancy 1, 2, 3 and/or 3 +

*MIP* malaria in pregnancy; *KABW* Knowledge, attitudes, and beliefs among women; *KABP* Knowledge, attitudes, and beliefs among providers; *SO* stockouts/availability of sulfadoxine-pyrimethamine

### Prevention of malaria during pregnancy

Information about the number of IPTp doses provided to pregnant women (e.g., IPTp coverage) varied greatly among the included documents; nine documents reported data on two IPTp doses (IPTp2); while only eight documents contained information about three or more doses (IPTp3 or IPTp3 +). Coverage of IPTp3 and IPTp3 + among women who had given birth in the previous 12 months ranged from 8.4% in a 2012–2013 nationwide evaluation [12] to 57% in one of the TIPTOP reports in 2020 [13].

Factors associated with IPTp2 uptake in one analysis conducted in 2014 among mothers of a child under the age of two included years of education, IPTp/ANC ideation, and exposure to malaria messaging [14]. Education was also found to be positively associated IPTp3 + uptake in a 2020 survey among women who had a pregnancy in the previous 12 months[15]. However, in another analysis, none of the assessed variables (i.e., age, socio-economic status, education), was associated with IPTp uptake [12]. Exposure to information, education and communication (IEC) campaigns was assessed, but no relationship was established between exposure to these campaigns and IPTp uptake or ITN use. In a secondary analysis of Demographic and Health Survey (DHS) and MIS data, in which researchers developed models to determine predictors of effective IPTp delivery, higher coverage was associated with increased education, living in an area with low to medium malaria transmission, seeking other maternal health services, and seeking care at public-sector health facilities [16]. A 2020 secondary analysis of MIS data confirmed the association between education and IPTp coverage, and also found that female-led households had higher IPTp uptake than male-led households [17].

Among pregnant women who reported having an ITN in their home in one survey, 98% reported sleeping under an ITN the previous night and understanding the benefits of ITNs for malaria prevention [18]. In this same survey, community members also reported prioritizing children and pregnant women in the household for ITN use.

### Care seeking among pregnant women

The reviewed documents contained data about care seeking (i.e., attending ANC, interacting with a CHV trained in MIP, or seeking treatment for fever or malaria) collected through cross-sectional surveys or from ANC registers.

In a 2017 survey, 89% of women reported attending at least one ANC visit during their most recent pregnancy [19]. This is consistent with data reported in the 2008–2009 DHS (91%) [20] and 2016 MIS (90%) [8]. The number of ANC visits during a previous pregnancy were more variable; ranging from 71% of women reporting a minimum of four ANC visits in one survey conducted in 2017 in areas with referral strengthening activities [18] to 47% in other DHS-led surveys [16].

Women reported different facilitators of and barriers to seeking care. Eight documents confirmed that barriers to care seeking and ANC attendance included distance from facilities; cost; negative perceptions of health facilities, including unwelcoming providers; a preference for traditional healers; a lack of personnel and supplies at health facilities; and/or concerns about personal security [14, 18, 19, 21–25]. For these reasons, women often self-treated or sought care from a traditional healer for fever or pregnancy, thereby delaying seeking care in a health facility. In a 2017 survey, women reported walking an average of 1.5 h to reach the health facility and then experienced long wait times upon arrival (an average of three to five hours) [25]. In another study, some women noted that CHVs, who are more accessible and less costly, were not providers, but health educators. As such, the pregnant women did not feel CHVs were well poised to provide care for febrile illness [19]. Other barriers were cultural in nature. Women noted that there were conditions caused by spiritual or cultural factors that HCWs were not trained to treat; these conditions required treatment by traditional healers [19]. Women also noted that they did not want to reveal their pregnancy beyond their immediate family until the second trimester, which prevented early ANC attendance [16].

A 2016 study found that the language used to describe malaria differed between providers and clients. For example, providers referred to malaria as *Tazomoka*, which translates to ‘mosquito fever’, whereas community members referred to it as *tazo*, which translates to ‘fever’; thus, the study concluded that health communication campaigns could be misunderstood [23].

Facilitators to care seeking were also reported, albeit less frequently. Some women mentioned being motivated to seek care because of the perceived high quality of care provided at facilities. In one study, most women of reproductive age (86.5%) felt confident in their ability to seek appropriate care [22]. A model which compared average estimates of ANC cost and delivery with average incomes did not identify cost as a barrier to ANC services [25], which was contrary to findings reported in other documents [19, 23].

### Quality of care

Eight documents reported on efforts to improve components of quality of care (e.g., provider knowledge and behaviour, availability of commodities) [4, 16, 19, 21, 26–29]. In one pilot survey in 2018, all 10 providers reported having had MIP training within the previous year. Yet, knowledge scores for IPTp and its correct administration were low, with only two providers (20%) correctly identifying the IPTp dosage schedule. Facility guidelines and materials were not in line with the most recent country guidelines at half (n = 5) of the facilities [24]. An evaluation of a toolkit designed for NMCP programme managers and HCWs to better understand MIP interventions and improve IPTp uptake found knowledge scores increased from 81 to 92% after receiving training on toolkit use [28].

A 2017 health facility assessment in Manajary District found providers possessed a high level of knowledge about malaria prevention and control; 100% cited ITN use and 94% cited SP as a means of preventing malaria. However, only two-thirds (64%) were comfortable assessing gestational age, a necessary action to early provision of IPTp. Only 11% cited malaria detection and treatment as a means of prevention and control [29].

Stockouts of SP were a major issue impacting quality of care in multiple documents. A 2015 report found that SP was available at 75% of university hospitals, 10% of regional referral and district hospitals, and 27% of health centres [21]. The documents reviewed reported on stockout rates for health centres more often than at secondary and tertiary levels (i.e., central and district hospitals); for health centres, this ranged from 11% [27] to 73% [21]. Notably, one report revealed that the National Central Pharmaceutical Warehouse was stocked out, affecting facilities’ ability to restock [27]. These reports focused on stock available on the day of the visit and did not include duration of stockouts. However, in one pilot survey, conducted at 10 health facilities in Moramanga District, some health staff reported no longer requesting SP because they assumed the district was stocked out [24]. Another rapid facility assessment conducted in 2017 in Manajary District found 64% of facilities citing stockouts as a major challenge. Additionally, over half (57%) of facilities did not possess an SP stock monitoring card so it was impossible to determine recent stockouts at those facilities [29].

None of the documents reviewed in the scoping exercise contained information about the treatment of malaria in pregnancy nor coordination needed between the NMCP and the MCH programmes or other relevant MOH divisions.

## Discussion

This scoping review highlights several common themes regarding prevention of malaria among pregnant women in Madagascar. Critical barriers to prevention activities and knowledge gaps were identified or confirmed by this review, and prevention measures with high reported uptake were noted across the studies, surveys and reports. The review confirmed the existence of few published studies on MIP in Madagascar and most available information was found in donor-funded project implementation reports (16 of the 23 documents). While many of the documents contained information on multiple elements considered essential for MIP programmes, none of the reviewed documents systematically reported on all the essential elements required for a comprehensive MIP programme; in particular, all were missing information about effective diagnosis and case management of MIP, as well as the collaboration needed between NMCP and MCH programmes. Collaboration with MCH is important because MIP activities are implemented on the ANC platform. Although the quality varied greatly, the studies and reports contained rich information to inform future intervention and research approaches.

### Patient-related factors

The most common barriers to MIP prevention included distance from health facilities [21], unwelcoming healthcare providers [3], and concerns about quality of care [19]. Some women preferred seeking care from traditional healers for fever or pregnancy care due to access or cultural beliefs [23, 30].

This review revealed that ITN use among pregnant women was consistently high, as was women’s knowledge of the importance of sleeping under an ITN. This is similar to findings from other malaria-endemic countries: a systematic review of 98 studies on IPTp and ITN use found that ITN delivery through ANC had fewer obstacles than IPTp uptake [31]. For IPTp coverage, however, data were challenging to compare because different doses of SP were collected over the years. This likely reflects reporting changes from when Madagascar adopted the WHO 2012 IPTp policy recommendations in 2014, and the updated policy in 2017. There was also variation in how the IPTp indicator was defined. For example, the MIS and DHS sampled women experiencing a live birth in the previous two years, whereas for the TIPTOP project, surveys included women who had ended a pregnancy (i.e., delivery, stillbirth, or spontaneous abortion) within the previous six months. Despite these variations, barriers to IPTp use in Madagascar were identified and included a lack of knowledge about IPTp and limited access to care due to distance. Concerns about IPTp safety and efficacy were not mentioned nor was the need for a husband’s permission as has been reported by other countries [32].

### System-related factors

This review identified health system weaknesses contributing to the quality of care such as commodity stockouts, the lack of data on supportive supervision of HCWs providing MIP services, and HCW knowledge gaps. Documents examining providers’ knowledge of MIP guidelines reported low competency in MIP interventions, and several partner reports planned for interventions to improve provider knowledge [21, 24, 28, 29]. These barriers to IPTp uptake are not unique to Madagascar; similar findings have been reported in other sub-Saharan African countries [30, 32, 33]. In Tanzania, for example, conflicting guidelines, insufficient time to deliver services, and out-of-pocket expenditures were barriers to care seeking among pregnant women [32]. In Nigeria, perceptions of poor quality of care and lack of HCW training for IPTp were obstacles to IPTp uptake [32]. A Zambian study found a lack of supervision and integration of services, in addition to drug shortages and long wait times, resulted in limited uptake of IPTp/ANC services [32]. Another systematic review found that local terminology for the disease was inconsistent with the biomedical terminology which affected care seeking behaviour [33]. This was also found in Madagascar [23].

### Gaps identified

Several gaps in reporting and implementation of activities to address barriers were identified. There is a dearth of high-quality, disaggregated data on malaria morbidity and mortality among pregnant women, with very little information on clinical management of pregnant women experiencing febrile illness. There is also a lack of information on potential solutions to increase IPTp uptake in Madagascar, such as utilizing traditional healers to provide targeted messages on MIP prevention or providing pregnant women with SP tablets at their first ANC visit to take at home each month instead of requiring directly observed therapy.

## Limitations

Relevant information in doctoral theses and reports from non-governmental organizations (NGOs) may have been missed in this review because they were not in searchable databases. However, informal consultations with key stakeholders were conducted to help mitigate this limitation. Databases from World Bank, Global Fund, UNICEF, and other partners were not searched, so it is likely that relevant documents were not reviewed for inclusion. Also, the quality of documents in the review varied; key variables were missing definitions, and statistical information for interpreting results or making comparisons was unavailable.

## Conclusion

This scoping review identified individual- and system-level deficiencies impacting the quality of MIP services in Madagascar. It identified available information and gaps (e.g., providing care closer to where women live); which gaps require more investigation (e.g., managing SP stockouts) and current interventions needing updates (e.g., ensuring MIP treatment/prevention are routinely included in supportive supervision of HCWs and CHVs). The lack of information about diagnosis and treatment of malaria among pregnant women and the coordination between NMCP and MCH are important programming considerations as well. Finally, the review identified noteworthy strategies requiring additional information, such as women’s perceptions of their access to IPTp or engaging traditional healers in delivering effective prevention messages. These findings can assist Ministries of Health and partners in strengthening comprehensive MIP programming efforts.

## Data Availability

Available upon request.
